# Passive training with upper extremity exoskeleton robot affects proprioceptive acuity and performance of motor learning

**DOI:** 10.1038/s41598-020-68711-x

**Published:** 2020-07-16

**Authors:** Shinya Chiyohara, Jun-ichiro Furukawa, Tomoyuki Noda, Jun Morimoto, Hiroshi Imamizu

**Affiliations:** 1 Brain Information Communication Research Laboratory Group, Advanced Telecommunications Research Institute International (ATR), Keihanna Science City, Kyoto, 619-0288 Japan; 2 Department of Psychology, Graduate School of Humanities and Sociology, The University of Tokyo, Hongo 7-3-1, Bunkyo, 113-0033 Japan; 3 Research Into Artifacts, Center for Engineering, School of Engineering, The University of Tokyo, Hongo 7-3-1, Bunkyo, 113-8656 Japan

**Keywords:** Neuroscience, Cognitive neuroscience, Perception

## Abstract

Sports trainers often grasp and move trainees’ limbs to give instructions on desired movements, and a merit of this passive training is the transferring of instructions via proprioceptive information. However, it remains unclear how passive training affects the proprioceptive system and improves learning. This study examined changes in proprioceptive acuity due to passive training to understand the underlying mechanisms of upper extremity training. Participants passively learned a trajectory of elbow-joint movement as per the instructions of a single-arm upper extremity exoskeleton robot, and the performance of the target movement and proprioceptive acuity were assessed before and after the training. We found that passive training improved both the reproduction performance and proprioceptive acuity. We did not identify a significant transfer of the training effect across arms, suggesting that the learning effect is specific to the joint space. Furthermore, we found a significant improvement in learning performance in another type of movement involving the trained elbow joint. These results suggest that participants form a representation of the target movement in the joint space during the passive training, and intensive use of proprioception improves proprioceptive acuity.

## Introduction

Sports instructors and physical therapists often ask their trainees to be in a passive state and physically move trainees’ limbs with their hands to instruct them on how to move the limbs. This “passive” training through physical contacts is one of the ways to help trainees to learn new skills and improve their motor performances efficiently. A merit of this training method is that a trainer can directly provide the trainee (learner) with proprioceptive information regarding the target joint. Proprioception is the primary sensory modality for the perception of body-states^1^, such as joint angles and muscle tension, and contributes to learning body movements^2–6^. However, to our knowledge, it is unclear if the learner’s attention to the instructed proprioceptive information is important for improvements in motor performance due to passive training. Here we hypothesised that changes in proprioceptive acuity due to passive training are a touchstone to examine whether learners attentively and intensively utilise the proprioceptive information during the training; accordingly, if learners use proprioceptive information to improve motor performances, their attention is directed to proprioception; as a result, proprioceptive acuity will increase after the training. Therefore, this study examined changes in proprioceptive acuity before and after the passive training and correlations between changes in the acuity and improvements in motor performances. Furthermore, to look at the effect of changes in proprioceptive acuity due to passive training on another type of movement involving the same joint, we examined whether changes in proprioceptive acuity of the elbow joint facilitate learning efficacy in a darts performance.

To this end, we used the exoskeleton robot^7,8^ instead of an end-effector robot. The exoskeleton is placed on the user’s entire body, just the upper or lower extremities, or even a specific body segment, such as the elbow or ankle. This device can apply forces independently at each joint, and therefore, it can directly guide the user’s posture (joint angle) in the joint space repeatedly. This advantage of the exoskeleton robot can repeatedly provide the user with an accurate sensory state of the desired movement at each joint.

A previous study^9^ demonstrated that proprioceptive training improves motor learning, and this study alternated a passive session, in which an end-effector robot instructed participants of a target movement, with an active session, in which the participants actively moved their arms; learning performances were higher when the passive session was alternated with the active session than when only the active sessions were employed. The findings suggest that participants use proprioceptive information to improve motor learning. However, because the passive training was alternated with active training, it was difficult to dissociate the effects of passive training from those of active training on motor learning. Therefore, we did not alternate the passive and active sessions, and instead, we asked participants to reproduce the instructed movement after the passive training.

Our study showed passive training improved not only the reproduction ability of the instructed movement but also proprioceptive acuity. Moreover, the improvement in proprioceptive acuity of the elbow joint facilitated learning efficacy in a darts performance. These results suggest that participants use proprioceptive information to represent the target movement in the joint space during passive training and improve motor performances, and the intensive use of proprioception during the training leads to an increase in proprioceptive acuity.

## Results

This study included 36 right-handed participants. We investigated the following three behavioural measures before and after the passive training of the elbow joint movement: (1) reproduction accuracy of the elbow movement, (2) proprioceptive acuity of the elbow joint, and (3) a learning performance in another type of movement involving the same joint (i.e., a dart performance).

### Passive learning of elbow joint movement

**Figure 1:**
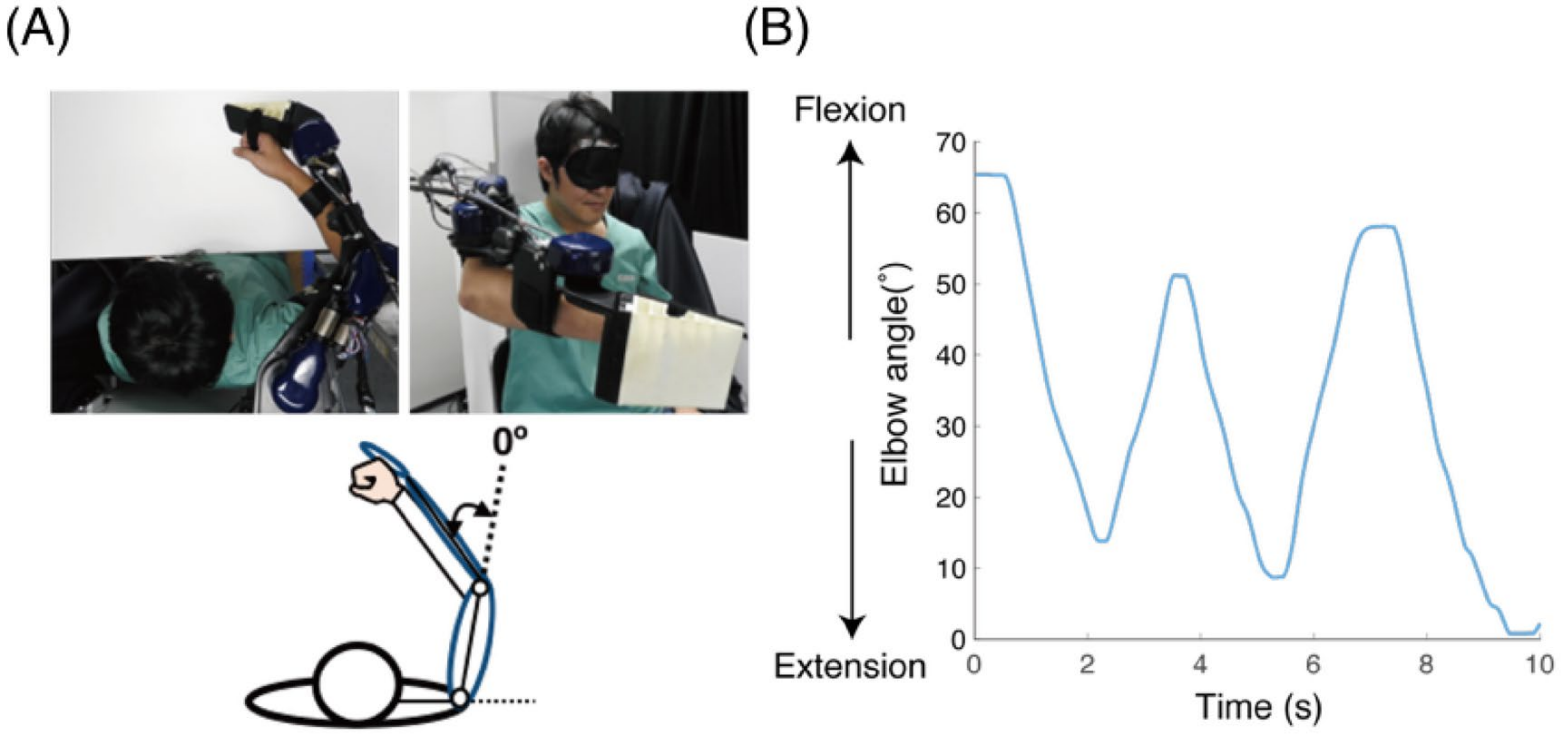
Experimental setup (**A**) and target trajectory (**B**). (**A**) An exoskeleton robot for passive instructions of flexion and extension movements of the elbow in the horizontal plane. The top view (the upper left photograph and the lower panel) and the right frontal view (the upper right photograph) of the exoskeleton robot. A parson in the photograph is the one of authors (not included in our participants) representing how participants’ arms attached to the robot. The eye mask occluded the participant’s vision during the trajectory instruction and its reproduction. (**B**) The target trajectory of the elbow joint. This trajectory was generated by an experimenter and was used for every trial and participant.

We used an exoskeleton robot (Fig. 1A) to instruct the participants of the target trajectory of the elbow joint. An exoskeleton-robot system, in which the limb is enclosed in an actuated robotic suit, can apply forces independently at each joint and configure the limb movements. The trajectory, which was generated by an experimenter, was a back and forth elbow movement for 10 s, as shown in Fig. 1B. This single trajectory was used for every trial and participant.

#### Trajectory learning session

**Figure 2:**
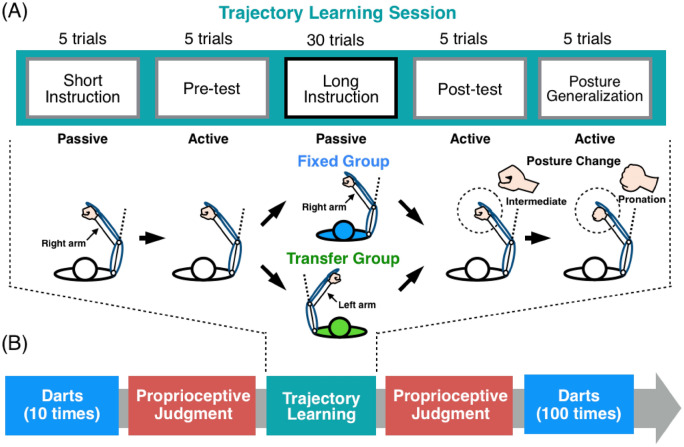
Experimental procedures. (**A**) Trajectory learning session. The main parts of this session included a long instruction period (the robot instructed participants of the target trajectory for 30 times) and a post-test session (the participants reproduced the instructed trajectory). Participants were divided into a fixed group (blue) and a transfer group (green) to test the transfer of the instruction across arms (the lower panels). Participants underwent the generalization-test to measure the generalisation of the instruction to different hand postures (the right panels). (**B**) The entire procedure. Participants underwent proprioceptive judgment sessions before and after the trajectory learning session to measure changes in proprioceptive acuity (red boxes). They participated in darts sessions at the beginning and end of the experiment to measure the influence of changes in proprioceptive acuity on another type of movement involving the same joint (cyan boxes).

The main part of our experiment was a trajectory learning session (Fig. 2A). The eye mask occluded the participant’s vision during the session. The session began with an instruction of the trajectory by the robot for five trials (short instruction) to instruct the target trajectory. Following this period, participants actively reproduced the target trajectory for five times in a pre-test to measure the baseline reproduction performance of each participant. Next, the robot instructed the trajectory for 30 times in a long instruction (learning) period, and participants then reproduced the trajectory for five times during a post-test period. We investigated individual improvements in trajectory reproduction performances from the pre-test to the post-test.

#### Transfer of instruction across arms

We investigated the transfer of the learning during the long instruction period across the arms to examine whether participants learn kinematic information in the proprioceptive space. All participants (*n* = 36) reproduced the trajectory using the right arm in the pre- and post-test periods (Fig. 2A). The target trajectory was instructed during the long instruction period to the right arm for one-half of the participants (Fixed Group: *n* = 18) and to the left arm for another half of the participants to investigate whether the instruction transfers across the arms (Transfer Group: *n* = 18). Notably, the target trajectory for the left arm was flipped around the sagittal plane from the trajectory for the right arm.

#### Generalisation of the learning effect to different hand postures

We investigated whether the participants preserve the learning effect on the right elbow joint even if the hand posture of the same arm changes. After the post-test period, participants changed their hand posture from the intermediate to the pronation position by inward rotating their forearms by 90°. The participants then reproduced the target trajectory five times in a posture generalisation period (Fig. 2A right).

#### Confirmation of passive movements

During the short and long instruction periods, participants were instructed to be in their passive states, i.e., being relaxed and not to contract any arm muscles. To confirm whether participants followed this instruction, electromyography (EMG) was conducted from the arm muscles: biceps (an agonist of the elbow flexion) and triceps (an agonist of the elbow extension). Participants were instructed to be relaxed during the long instruction period and to actively reproduce the trajectory during the post-test period. The EMG amplitudes were compared between these two periods, and the amplitude was averaged across participants. As a result, the amplitude in the instruction period was significantly smaller than that in the post-test period [biceps; *t*(17) = 5.64, *p* < 0.001, triceps; *t*(17) = 3.23, *p* < 0.005]. These results indicate that participants are in their passive state during the long instruction period (see SI Fig. S1 online for EMG traces and the average EMG activity levels).

#### Results of trajectory learning

**Figure 3:**
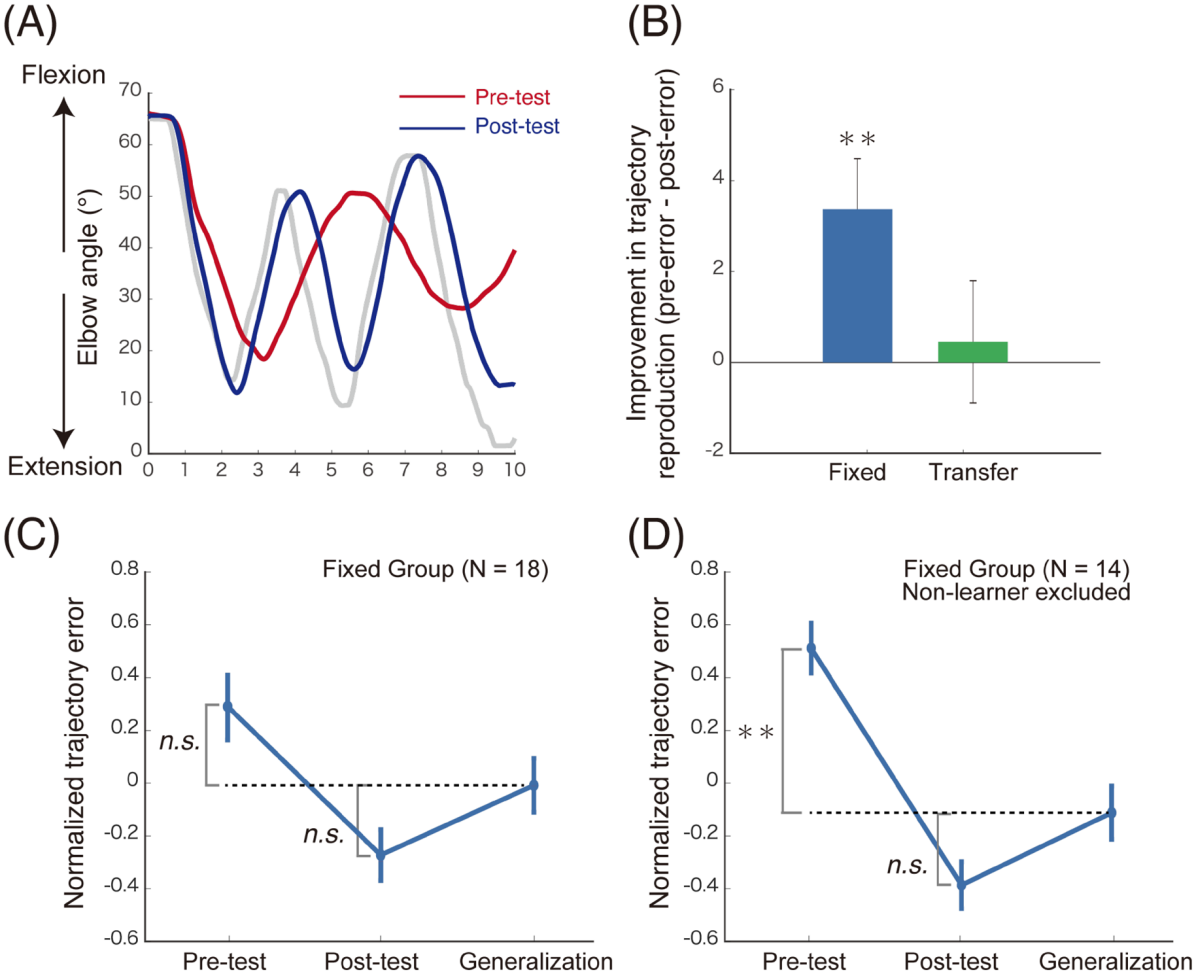
Findings in the trajectory learning session. (**A**) The target trajectory of the elbow joint (grey trace) and trajectories reproduced by a representative participant. Reproduced trajectories were averaged within a pre-test (red trace) or a post-test period (blue trace). (**B**) Index of improvements in trajectory reproduction performance measured by subtraction of the error in the post-test period from the error in the pre-test period. The error decreased significantly from the pre-test to the post-test period in the fixed group (blue bar), but not in the transfer group (green bar). (**C**) Trajectory reproduction errors in the pre- and post-tests as well as a generalization-test to a different hand posture in the fixed group. The reproduction error was normalised across the three tests for each participant. (**D**) Trajectory reproduction errors when the four non-learners (see main texts for definition) were excluded from the data represented in (**C**). Error bars represent standard errors. *p < 0.05, **p < 0.01; *n.s*. not significant.

Figure 3A shows reproduced trajectories of a participant in the fixed group averaged across trials during the pre-test (red) and post-test (blue). We calculated a trajectory error as root mean square error between the target trajectory and the trajectory reproduced by the participants. Then, we subtracted the error in the post-test period from the error in the pre-test period to investigate improvements in the trajectory reproduction performance (Fig. 3B). We found a significant decrease in the error from the pre-test period to the post-test period in the fixed group [a paired test: *t*(17) = 3.139, *p* = 0.006]. In contrast, we did not find a significant change in the trajectory in the transfer group [a paired test: *t*(17) = 0.346, *p* = 0.734]. These results suggest that there is a significant learning effect during the long instruction period, and the movement instruction given to the left arm by the robot hardly transfers to the right arm.

#### Generalisation of learning effects to different hand postures

In the fixed group, we normalised the error across the three periods for each participant to investigate the individual differences in the baseline error. We predicted that we would obtain either of the following two results: if the learning effect is generalised to different hand postures, the error in the posture generalisation period is significantly smaller than that in the pre-test but does not significantly change from the error in the post-test; in contrast, if the effect is not generalised, the error in the generalisation period is not significantly different from that in the pre-test and greater than that in the post-test. However, the error in the generalization period was not significantly different from that in the pre-test [a paired test: *t*(17) = − 1.47, *p* = 0.320 Bonferroni corrected] or the post-test [*t*(17) = 1.76, *p* = 0.192 corrected; Fig. 3C]. Then, we excluded participants (*n* = 4) whose errors did not decrease from the pre-test to the post-test (non-learners, [average error in the pre-test] ≤ [average error in the post-test]) and reanalysed data of the remaining participants (*n* = 14). Consequently, the error in the generalization period was significantly smaller than that in the pre-test [*t*(13) = − 3.74, *p* = 0.005 corrected] but not significantly different from that in the post-test [*t*(13) = 1.72, *p* = 0.220 corrected; Fig. 3D]. These results suggest that the training effect is generalised to different hand postures (pronation posture) if participants successfully learn a target trajectory in a posture (intermediate posture). We excluded the four non-learners from the fixed group in further analyses. Note that we found a significant improvement in the trajectory reproduction in the fixed group (Fig. 3B), even when the non-learners were excluded (see SI Fig. S2 online).

### Proprioceptive-acuity change

**Figure 4:**
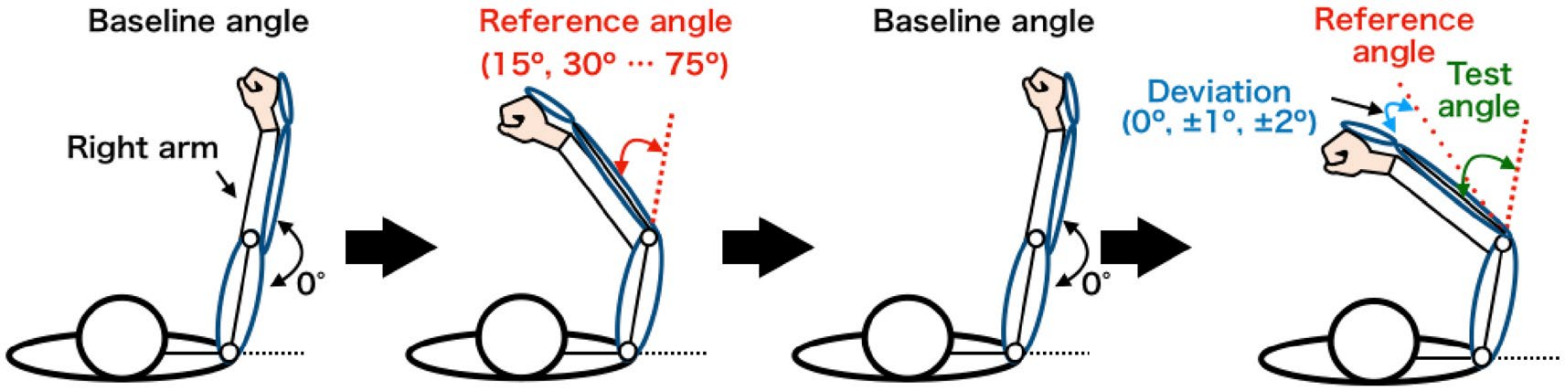
Procedure in the proprioceptive judgment trial. At the beginning of each trial, participants extended their elbow joint to the baseline angle (angle = 0°). The robot flexed the participant’s right elbow joint from the baseline angle to a reference angle, which was pseudo-randomly selected from 15°, 30°, 45°, 60°, or 75°. Then, the robot returned the arm to the baseline angle and again flexed the arm to a test angle. The test angle deviated from the previous reference angle by a subtle angle that was pseudo-randomly selected from 0°, ± 1°, and ± 2°. Participants judged whether the test angle was smaller than, the same as, or larger than the reference angle.

We examined individual proprioceptive acuity before and after the trajectory learning session (red boxes in Fig. 2B). The eye mask also occluded participants’ vision in this session. This session consisted of 30 trials. At the beginning of each trial, participants extended their elbow joint to the baseline angle (angle = 0°: Fig. 4). The robot flexed the participant’s right forearm from the baseline angle to a reference angle, which was pseudo-randomly selected from 15°, 30°, 45°, 60°, or 75° for each trial. Participants were asked to be in the passive state (see above) and to remember the reference angle. Then, the robot returned the arm to the baseline angle and flexed the arm to a test angle. The test angle deviated from the previous reference angle by a subtle angle that was pseudo-randomly selected from 0°, ± 1°, and ± 2° for each trial. Participants verbally reported whether the test angle was smaller than, the same as, or larger than the reference angle. We defined a proprioceptive acuity as the ratio of the number of the correct response (− 1° or − 2°: “smaller”; 0°: “same”; 1° or 2°: “larger”) to the total responses (30 trials). We calculated individual index of improvements in proprioceptive acuity by subtraction of the acuity before the trajectory leaning session from the acuity after the session.

**Figure 5:**
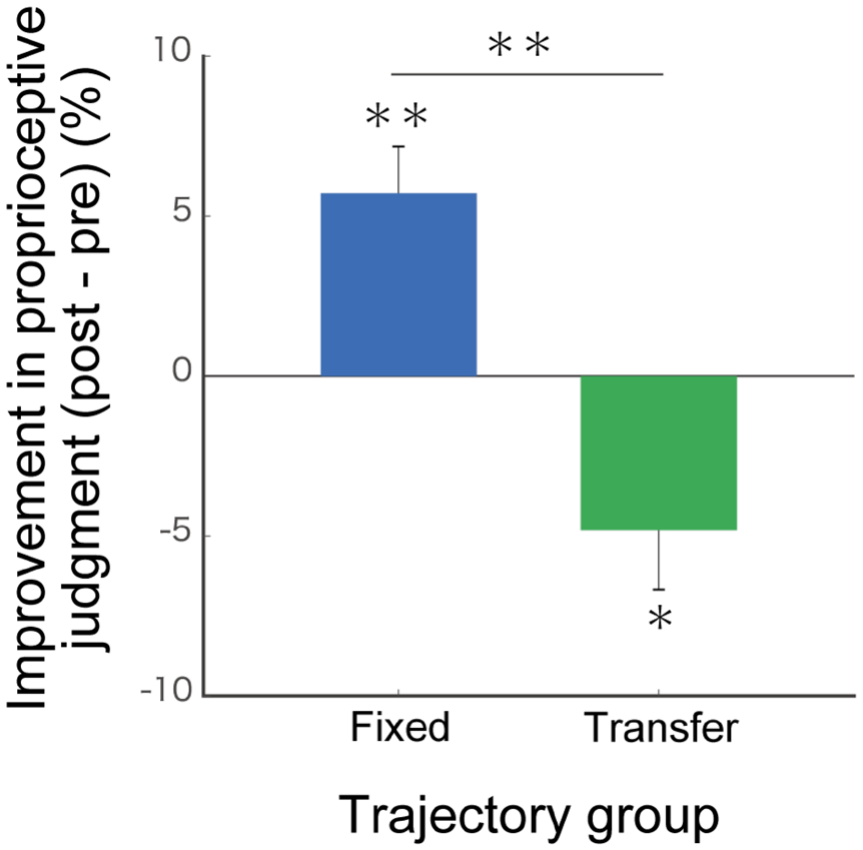
Improvement in proprioceptive acuity and its correlation with improvement in trajectory reproduction. A significant improvement was observed in the fixed group (blue bar) but not in the transfer group (green bar). *p < 0.05, **p < 0.01.

Figure 5 shows the index of improvements in the proprioceptive acuity in both groups. We found a significant improvement in the fixed group [a paired test: *t*(13) = 3.92, *p* = 0.002]. In contrast, we did not find an improvement but observed a significant decrease in the transfer group [a paired test: *t*(17) = − 2.60, *p* = 0.019]. There was a significant difference in the index of improvement between the two groups [two-sample *t* test, *t*(30) = 4.27, *p* < 0.001]. These results indicate that the proprioceptive acuity improves after the repeated instruction by the robot, and the improvement is specific to the instructed arm. In short, learning the elbow joint movement by the passive instruction is associated with the improvement in proprioceptive acuity of the joint angle.

### Influence on darts performances

**Figure 6:**
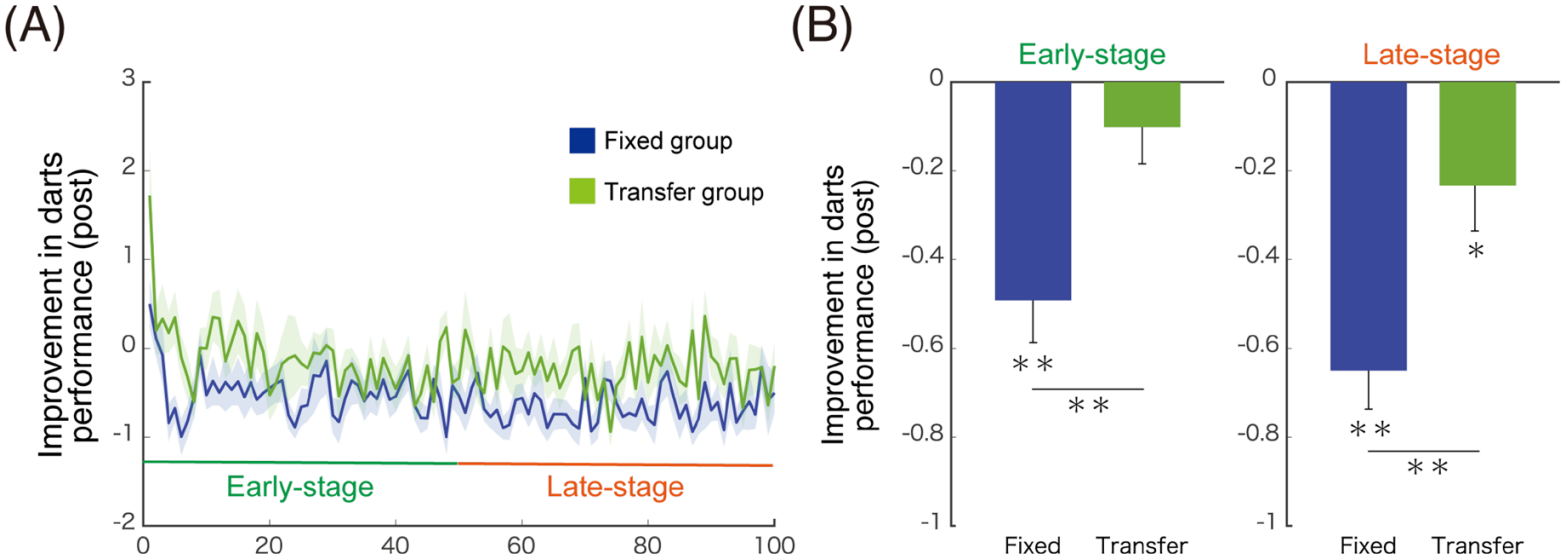
Generalisation of elbow-joint training to darts performances. (**A**) Post-session data of darts performances calculated as z score (mean and standard deviation) in pre-session. Plots are mean ± SE (standard error) across participants in fixed (blue) and transfer (green) groups. (**B**) Early-stage improvement (averaged across 1st–50th trials in the post-session) and late-stage improvement (averaged across 51st–100th trials) in darts performances. A significant early-stage improvement was observed only in the fixed group, and a significant late-stage improvement was observed in both groups. The improvement in the fixed group was significantly greater than that in the transfer groups at the early- and late-stages. *p < 0.05, **p < 0.01.

We investigated whether the effects of passive elbow-joint training facilitate the performance in another type of movement (i.e., darts throwing) involving the same joint. Participants underwent a darts session before and after the experimental procedures explained (cyan boxes in Fig. 2B). The participants threw a darts aiming at the centre of a target board using their right arms. The first (pre-) and the second (post-) sessions consisted of 10 and 100 times (trials) of throwing, respectively. A darts error was measured by the unsigned distance between the darts-tip and the centre of the target board. The post-session data were normalised as z scores using the mean and standard deviation in pre-session for each participant. We investigated improvements in darts performances from the pre-session to the post-session separately at two stages of the learning. One is the early-stage improvement measured as the average normalised-error across the early trials (1st–50th) of the post-session, and the other is the late-stage improvement measured as the average normalized-error across the late trials (51st–100th) of the post-session (Fig. 6A).

We found a significant early-stage improvement (Fig. 6B, left panel) in the fixed group [a paired test: *t*(13) = − 5.31, *p* < 0.001] but not in the transfer group [*t*(17) = − 1.21, *p* = 0.242)]. We observed a significant late-stage improvement (Fig. 6B, right panel) in the fixed group [a paired test: *t*(13) = − 8.17, *p* < 0.001] as well as in the transfer group [*t*(17) =  − 2.35, *p* = 0.031]. The improvement in the fixed group was significantly greater than that in the transfer group at the early- and late-stages (early-stage: *p* = 0.004; late-stage: *p* = 0.004).

## Discussion

This study investigated how passive training contributes to motor learning. Specifically, we examined (1) generalisation of passive training on a specific joint (the right elbow joint) to the opposite arm and a different hand posture, (2) changes in proprioceptive acuity after the passive training, and (3) effects of changes in proprioceptive acuity on learning performances of another type of movement. Our results showed that passive training improved the performance of the trajectory reproduction and the proprioceptive acuity. The performance improvement was generalised to a different hand posture of the same arm but not to the hand posture of the opposite arm. We also found that the improvement in the proprioceptive acuity enhanced the performance of another type of movement involving the same joint (i.e., learning performance in a darts). The present findings suggest that participants form a representation of the target movement in the joint space during the passive training, and intensive use of proprioception improves the proprioceptive acuity.

We could not find a significant generalisation of improvement in the trajectory reproduction across the arms (Fig. 3B: a transfer group). This result suggests that participants learned and represented the target trajectory based on the intrinsic information (such as proprioception and muscle length), which is specific to the trained joint and arm. This finding is consistent with the improvement in proprioceptive acuity due to the passive training (Fig. 5). We found a significant generalisation of the improvement in trajectory reproduction to different hand postures (Fig. 3D). This result suggests that the learned representation of the target trajectory is independent of the hand posture.

Participants in the transfer group did not undergo a post-test with the left arm. A reason is that it is necessary to require participants to actively reproduce the trajectory with the left arm before the post-test with the right arm. It is known that the effect of the active training transfers from one arm to the opposite arm (intermanual transfer)^10–14^. Therefore, our study was designed not to reproduce the trajectory using the left arm to avoid the possibility that active movement using the left arm may affect the performance of the right arm in the post-test. It is unlikely that the learning performance of the left arm is significantly different from that of the right arm for the elbow joint movement. Thus, the participants in the transfer and fixed groups might learn the trajectory at similar degrees.

We identified an improvement in proprioceptive acuity in the fixed group but not in the transfer group. A possible reason for the acuity improvement is that the passive training required the attention of the participants to the proprioceptive information on the trained joint. Several studies demonstrated that attention to a specific sensory modality enhances its sensitivity and acuity^15–21^. Thus, the proprioceptive attention may have improved the sensory acuity and affected the reproduction performance of the target trajectory. Our results are consistent with those in a previous study, which showed that proprioceptive acuity to a target position improved after the repetition of a passive reaching movement to the position^22^. Furthermore, our proprioceptive judgment task examined changes in the acuity at multiple positions included in the target trajectory (Fig. 4). The results of the judgment task indicate that passive training improves the acuity not only at a specific position but also at positions included in the trained trajectory.

We investigated if the passive training on a specific joint affects the learning performance of another type of movement. We found that improvement in darts performance was more significant in the fixed group than in the transfer (control) group (Fig. 6). These results suggest that the passive training on a specific joint improves its proprioceptive acuity and enhances the learning of the darts performance at the same joint. Moreover, we identified a significant improvement only for the fixed group but not for the transfer group in the early stage (Fig. 6B). This result suggests that the enhancement is prominent in the early stage of learning. Many studies have demonstrated that uncertainty in sensory information slows down the rate of motor learning and adaptation^23–26^. A previous study found a significant correlation between variability in proprioceptive acuity and slow rates of adaptation to visuomotor rotation in elderly people^27^. In our study, the improvement in proprioceptive acuity after the passive training probably reduced uncertainty or variability in proprioceptive information on the elbow joint. As a result, the rate of the learning was increased in dart throwing performance at the same joint in the fixed group and the transfer group.

In conclusion, the present study indicates that passive training improves proprioceptive acuity around the trained joint and learning performance of another type of movement involving the same joint. The findings of this study may help understand a role of proprioceptive information during passive training and efficient training method. We examined the effects of passive training on a single joint. However, people often use passive instruction based on physical contacts during the training of the complex multi-joint movement. Therefore, further study is needed to investigate the effects of passive training on a multi-joint movement. We also did not measure proprioceptive acuity at different posture and joint variability at dart throwing, and therefore, it is difficult to understand mechanisms underlying improvements of the learning performance of another type of movement. Future modelling and empirical studies are also required to investigate mechanisms underlying improvements in proprioceptive acuity and enhancement of the learning performance for another type of movement. Such studies would help understand mechanisms underlying supporting passive training by an exoskeleton robot, which is an emerging training device in sports and rehabilitation.

## Methods

### Participants

We recruited healthy participants in their twenties to avoid the effects of aging and disease on their proprioceptive acuity. We also recruited right-handed participants to control for the effects of handedness on elbow movements and darts performance. We selected participants who were novice to darts, i.e., they reported that they had thrown darts less than three times in their life, to investigate learning in darts performance. A total of 36 right-handed individuals (24 males and 12 females, aged 22–29) without known histories of visual, neurological, or musculoskeletal disfunctions participated in this study. We randomly assigned the 36 participants to either the fixed group (*n* = 18) or transfer group (*n* = 18). We determined the sample size of each group according to previous studies on passive motor learning (*n* = 11 or 12 per participant group)^9^ and proprioceptive learning (*n* = 14 per group)^3^. The participants gave written informed consent prior to the experiment. All experiments were approved by the ethics committee of the Advanced Telecommunications Research Institute International, Kyoto, Japan and were conducted according to the Declaration of Helsinki.

### Experimental apparatus

#### Exoskeleton robot

We used the upper limb exoskeleton robot in this study^7,8^. The link lengths are 0.265 m from the shoulder to the elbow joint and 0.26 m from the elbow to the wrist, and the upper limb exoskeleton robot has four degrees-of-freedom: shoulder flexion/extension, shoulder abduction/adduction, elbow flexion/extension, and wrist flexion/extension joints. In this study, only the elbow flexion/extension joint was moved by an electric actuator during the instruction of the target trajectory, while the shoulder and wrist joints were fixed with a pin. The participants’ arms were attached to the exoskeleton arm by Velcro straps, and the participants were asked to lightly grip the hand strap but not to change their finger positions. The robot was programmed to guide the participant’s arm to the start position during all the trials. Four seconds after arriving at the start position, a start cue of the movement was given to the participants. The elbow joint movement was then actively or passively performed, and an end cue was given to the participants 10 s after the start cue.

#### Electromyography

We used EMG to measure muscle activities during the passive instruction of the target trajectory and its active reproduction. The EMG signal was measured from the major muscles (the biceps and triceps) spanning the elbow joint. Ag/AgCl bipolar surface electrodes were attached on the muscle belly after abrasion of the skin.

#### Darts

A ‘hard dart’ board and steel tip darts (Puma darts production Ltd.; https://www.shotdarts.com) were used in this study. The dartboard was set up in accordance with international rules: the centre of the board was 1.73 m above the floor, and the face of the board was placed at a distance of 2.37 m from the throw line.

### Data analysis

#### EMG analysis

The EMG signal was high-pass filtered using a fourth-order Butterworth filter with a 10 Hz cutoff and full-wave rectified. The filtered and rectified signal was smoothed by scaling the EMG amplitude at each time point by the root mean square of the signal in a 100-ms window centred at that time point. Following this preprocessing, we performed a within-participant, within-muscle normalisation of each EMG trace by dividing the EMG amplitude at each time point by the maximum EMG amplitude produced during the all active conditions (i.e., the pre-test, post-test, and posture generalization-test) (% MVC: percentage maximum voluntary contraction). We compared EMG of the agonist muscle during the post-test period (active movement) with that during the long instruction period (passive movement) for each muscle (biceps or triceps). We determined the flexion and extension periods according to the target trajectory and extracted EMG data in each period from the recorded data. We calculated each muscle activity when it was used as the agonist muscle (the biceps are the agonist in the flexion period or in the extension period). We averaged normalised EMG amplitude within each period across trials and participants.

#### Trajectory performance analysis

We measured the angle of the elbow joint at 250 Hz with an encoder of the exoskeleton robot. We computed root mean square error between the target and the reproduced trajectories of the elbow joint. We used a paired *t* test to compare the errors between the pre- and post-tests separately for each group to investigate the learning of trajectory instructed passively. The error was normalised as z score across the three test-periods for each participant to examine generalisation across the hand postures. We used a paired *t* test to compare the errors in the pre-test with those in the posture-generalization test or the errors in the post-test with those in the posture-generalization test in the fixed group. We then excluded non-learners whose average number of errors did not decrease from pre-test to post-test ([average error in the pre-test] ≤ [average error in the post-test]) in the fixed group and reanalysed the data of the remaining participants.

#### Perceptual judgments analysis

We calculated the index of improvements in proprioceptive acuity (see the main text for definition) for each participant by subtraction of the acuity in the pre-session from the acuity in the post-session. We applied a paired *t* test to the acuity in pre- and post-session to evaluate a statistical significance of the improvement in proprioception.

#### Darts performance analysis

Because we recruited novice participants for dart throwing, their initial performances were largely different among participants. Thus, the post-session errors were normalised as z scores using the mean and standard deviation in the pre-session within each participant. We applied a paired *t* test to compare the error in the pre-session with that in the early post-session (1st–50th trials: early-stage improvement) or the late post-session (51st–100th trials: late-stage improvement). We applied a two-sample *t* test to investigate differences in the normalised error (see above) between the fixed and transfer groups.

## Supplementary information

Supplementary file1 (DOCX 288 kb)
