# Supplementary material for: Passive training with upper extremity exoskeleton robot affects proprioceptive acuity and performance of motor learning
Source: Sci Rep. 2020 Jul 16;10:11820. doi: 10.1038/s41598-020-68711-x (PMC7366915; doi:10.1038/s41598-020-68711-x)
Supplement: Supplementary file 1 — Supplementary file1 (DOCX 288 kb) [file 41598_2020_68711_MOESM1_ESM.docx]

Supplementary Information

**Passive Training with Upper Extremity Exoskeleton Robot Affects Proprioceptive Acuity and Performance of Motor Learning**

Shinya Chiyohara, Jun-ichiro Furukawa, Tomoyuki Noda, Jun Morimoto^, *^, and Hiroshi Imamizu

Correspondence to J.M. (email: [xmorimo@atr.jp](mailto:xmorimo@atr.jp))

SI Figures


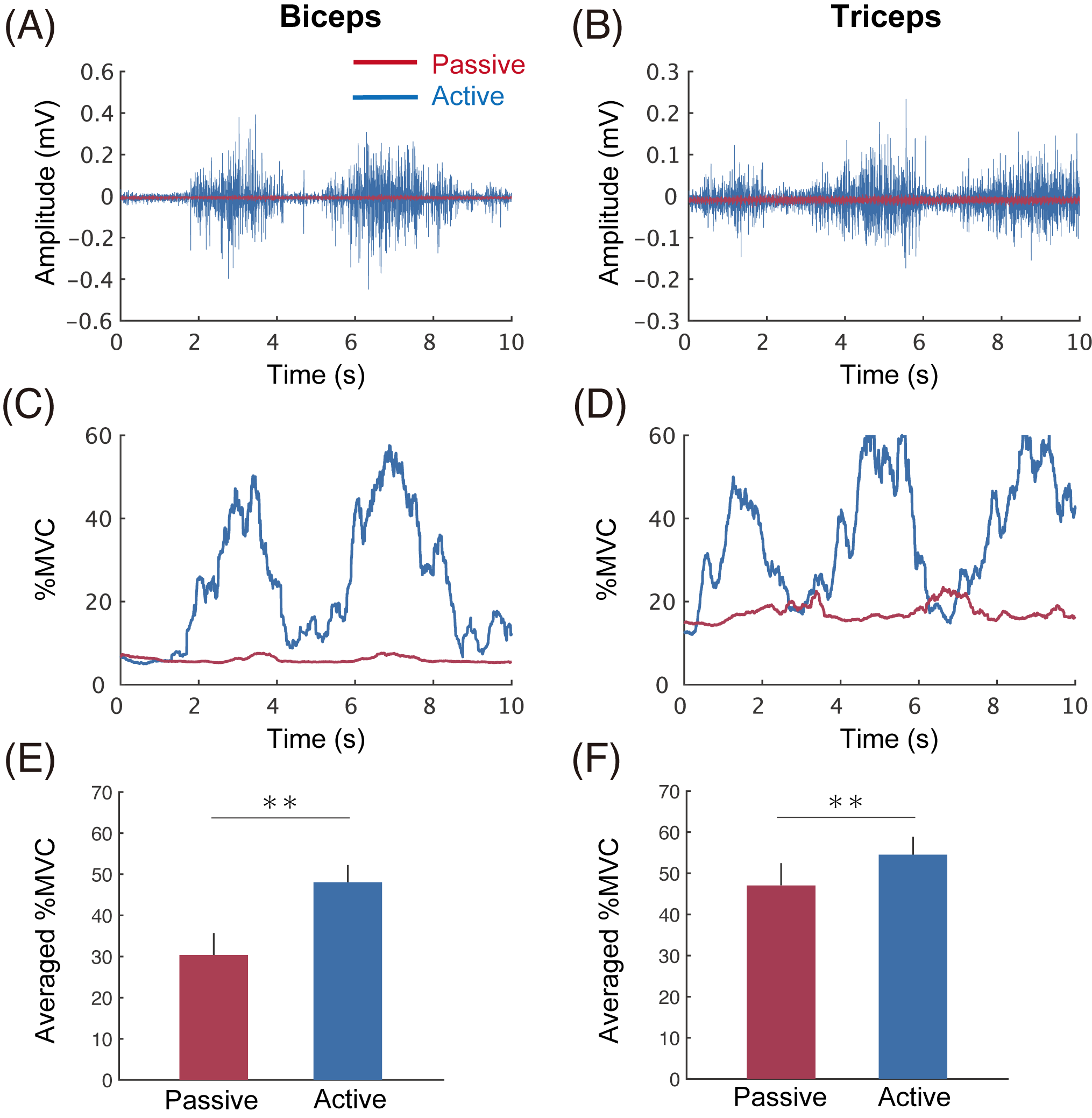


**Supplementary Figure S1.** EMG activity during passive (red) and active (blue) conditions. A representative participant’s EMG signals in biceps (A) and triceps (B). EMG activity level of the same participant (%MVC) in biceps (C) and triceps (D). EMG activity levels averaged across participants in the fixed group in biceps (E) and triceps (F). Error bars represent standard errors. ** *p* < 0.01


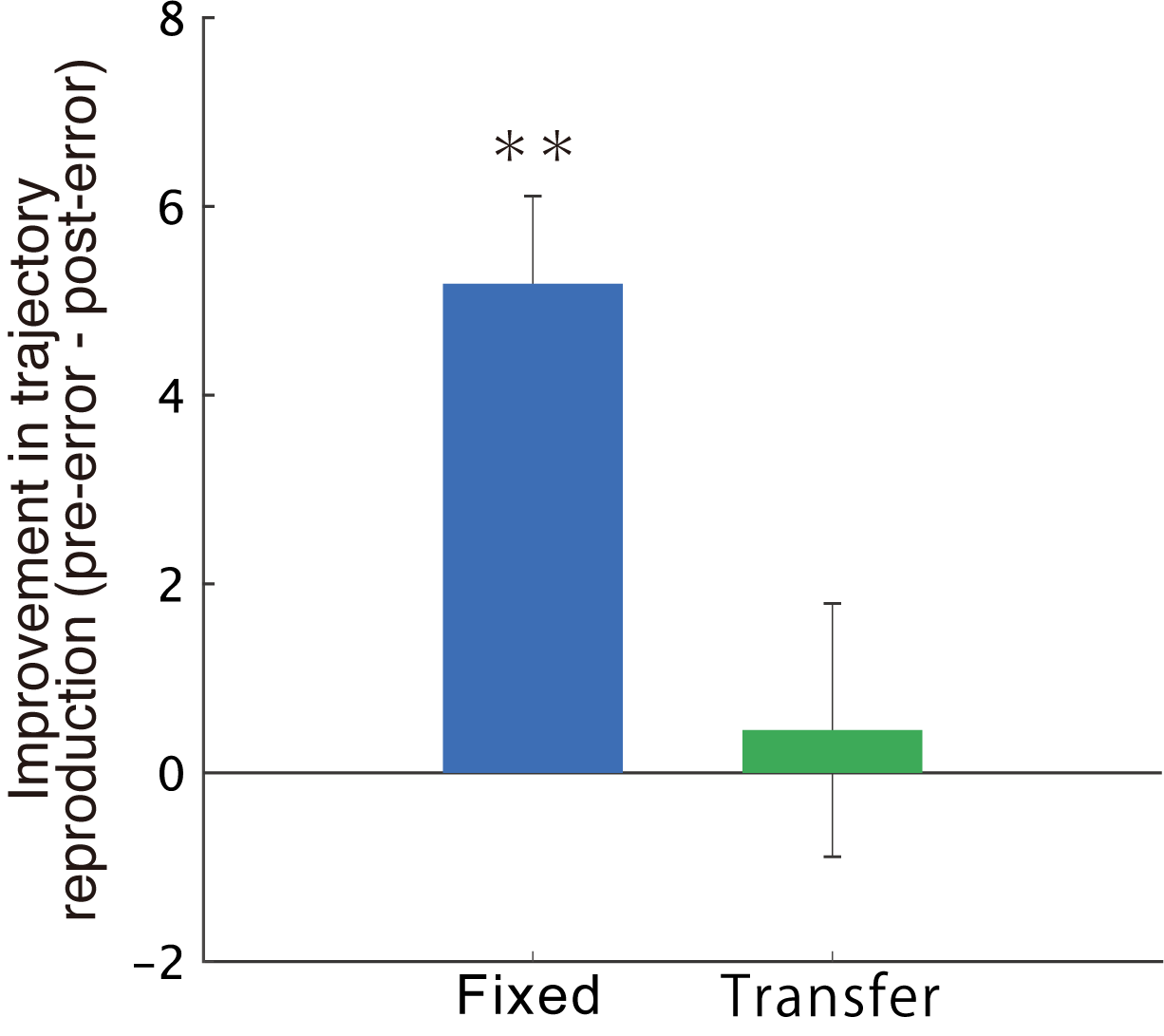


**Supplementary Figure S2.** Index of improvements in trajectory reproduction performance when the four non-learners (see main texts for definition) were excluded from the data. The error decreased significantly from the pre-test to the post-test period in the fixed group (blue bar) [a paired test: *t*(13) = 5.778, *p* = 0.001] but not in the transfer group (green bar). Error bars represent standard errors. ** *p* < 0.01
